# Rapid divergent coevolution of *Sinopotamon* freshwater crab genitalia facilitates a burst of species diversification

**DOI:** 10.1111/1749-4877.12424

**Published:** 2020-04-22

**Authors:** Fengxin YAO, Boyang SHI, Xiaoqi WANG, Da PAN, Ming BAI, Jie YAN, Neil CUMBERLIDGE, Hongying SUN

**Affiliations:** ^1^ Jiangsu Key Laboratory for Biodiversity and Biotechnology, College of Life Sciences Nanjing Normal University Nanjing China; ^2^ Key Laboratory of Zoological Systematics and Evolution, Institute of Zoology Chinese Academy of Sciences Beijing China; ^3^ Department of Biology Northern Michigan University, Marquette Michigan USA

**Keywords:** coevolution, genital diversification, freshwater crab, morphological evolution

## Abstract

One of the most striking radiations in brachyuran evolution is the considerable morphological diversification of the external reproductive structures of primary freshwater crabs: the male first gonopod (G1) and the female vulva (FV). However, the lack of quantitative studies, especially the lack of data on female genitalia, has seriously limited our understanding of genital evolution in these lineages. Here we examined 69 species of the large Chinese potamid freshwater crab genus *Sinopotamon* Bott, 1967 (more than 80% of the described species). We used a landmark‐based geometric morphometric approach to analyze variation in the shape of the G1 and FV, and to compare the relative degree of variability of the genitalia with non‐reproductive structures (the third maxillipeds). We found rapid divergent evolution of the genitalia among species of *Sinopotamon* when compared to non‐reproductive traits. In addition, the reconstruction of ancestral groundplans, together with plotting analyses, indicated that the FV show the most rapid divergence, and that changes in FV traits correlate with changes in G1 traits. Here we provide new evidence for coevolution between the male and female external genitalia of *Sinopotamon* that has likely contributed to rapid divergent evolution and an associated burst of speciation in this lineage.

### INTRODUCTION

Understanding the mechanisms that contribute to the spectacular diversification of living things has long interested evolutionary biologists (Darwin 1871; Eberhard 1985; Panhuis *et al.* 2001; Hosken & Stockley 2004; Kraaijeveld 2011; Ah-King *et al.* 2014). The evolutionary processes that govern the genital divergence of animals are still among the most intriguing questions about the mechanisms of diversification (Eberhard 1985; Arnqvist 1997; Hosken & Stockley 2004). Several evolutionary hypotheses have been proposed to explain genital divergence (Arnqvist 1998; Hosken & Stockley 2004; Eberhard 2010). Recent theoretical and empirical studies support sexual selection as a key driver of genital diversification and coevolution in a number of groups (Holland & Rice 1999; Briceño & Eberhard 2009; Pizzari & Snook 2010; Maan & Seehausen 2011; Simmons & Garciagonzalez 2011; Dougherty *et al.* 2017). Sexual selection has generally been considered to be a positive diversifying force driving the evolution of genitalia and increases in the rate of speciation (Darwin 1871; Panhuis *et al.* 2001; Hosken & Stockley 2004). Recent studies have contributed to our understanding of divergence and coevolution in animal genitalia (Eberhard 2010; Masly 2012; Hopwood *et al.* 2016), but most of those works have lacked data on female genitalia (Eberhard 1985, 2004). However, the exact mechanisms that have promoted the rapid genital divergence in many groups (including the freshwater crabs) remain debatable and are still mostly unknown. This is especially true when the processes of species diversification are considered within temporal and spatial frameworks.

The external reproductive structures of brachyurans (the male first gonopod [G1] and the female vulva [FV]) are typically complex structures that exhibit considerable morphological variation between species. For example, in the case of male primary freshwater crabs (hereinafter referred to as freshwater crabs) the unique shape of the G1 is used as a high-weight species-level character in the taxonomic identification of species in every family (Cumberlidge 1999; Dai 1999; Chen *et al.* 2007; Ng *et al.* 2008; Guinot *et al.* 2013). The remarkably high morphological diversification of the male G1 of freshwater crabs is one of the most striking evolutionary radiations seen in any group of brachyurans, but the exact mechanisms producing this diversity have long been a topic of discussion for carcinologists and evolutionary biologists (Cumberlidge 1999; Dai 1999; Guinot *et al.* 2013; Davie *et al.* 2015). Freshwater crabs are heterotremes that have complicated genital structures. The males have 2 pairs of abdominal pleopods or gonopods (G1 and G2), while females have a pair of genital openings on sternite 6 of the thoracic sternum, known as the FV. The external genitalia of both sexes of freshwater crabs are located in the sternopleonal cavity of the thoracic sternum and are covered by the folded pleon except when mating is occurring (Brandis *et al.* 1999; Dai 1999; Klaus & Türkay 2014; Chu *et al.* 2017a,b, 2018). Although Dai (1999) illustrated the FV for a large part of the Chinese freshwater crab fauna, she did not employ them as taxonomic characters that could be used to distinguish between species and genera. The external features of the FV comprise a hardened calcified rim surrounding the vulval opening (Dai 1999). The detailed morphology of this external structure varies with species and is important for successful mating, and differences here may reflect the morphological changes taking place during the adaptive evolution of the genitalia (Brandis *et al*. 1999). Other changes in the softer non-calcified reproductive structures that lie deep to the FV openings are largely unknown (Brandis *et al*. 1999). The correspondence between the morphology of the male and female genitalia of freshwater crabs and its role in successful copulation has been noted by Pretzmann (1973) and reviewed in Guinot *et al.* (2013). The first and second male gonopods (G1 and G2) of potamid freshwater crabs function together during copulation as intromittent organs that deliver spermatophores from the penis (a small papilla at the end of the vas deferens positioned at the base of the coxa of each pereiopod 5) to the female openings or FV on the left and right sides of thoracic sternite 6. During mating, the tip of the G1 terminal article is inserted into the outer opening of the FV but does not penetrate further, while the flexible tube-like G2 terminal article reaches deeper inside the vagina to deliver spermatophores to the female's spermathecal (Brandis *et al*. 1999). The G1 has a species-specific morphology that makes it useful in taxonomy, and this is the reason that it is the focus of the present study. In contrast, the G2 was not selected for study here because it has a conservative structure that is similar in all species of *Sinopotamon* (Klaus *et al.* 2009).

Bott (1970) argued that because the male and female genitalia are normally covered by the tight-fitting pleon, they are less affected by external selective forces arising from environmental changes compared to other more exposed external morphological traits (Türkay 1975). A perfect complementary match between the shape of the male and female genitalia in brachyurans is thought to be necessary for successful copulation and sperm transfer, which means that these structures in males and females have coevolved under natural selection (Guinot 1979, 2013). Freshwater crabs normally mate when both sexes are in intermolt and are hard-shelled, which ensures that the G1 and G2 are rigid and capable of reaching the FV and of bringing about inseminating the female (Liu & Li 2000; Guinot 2013). Freshwater crabs are a particularly diverse group for studying the correspondence between the characters of the male and female genitalia, and the role that these structures play during copulation (Pretzmann 1973; Brandis *et al.* 1999; reviewed in Guinot *et al.* 2013). Despite this, very little is known regarding the evolution of the male and female genitalia in brachyurans, including the freshwater crabs. The few quantitative studies that are available have focused mostly on the male genitalia, and the corresponding characters of the females have been largely overlooked.

*Sinopotamon* is the most species-rich genus of freshwater crabs in China and is the group with the largest geographic distributional range in this global biodiversity hotspot (Dai 1999; Yeo *et al.* 2008; Cumberlidge *et al.* 2009, 2011; Fang *et al.* 2013; Chu *et al.* 2018). The striking morphological variation of the male G1 in species of *Sinopotamon* has been used as a high-weight character in systematic classification (Dai 1999; Chu *et al.* 2018). In contrast, the external structure of the FV was formerly considered to be relatively simple and invariable, and, consequently, has been overlooked in taxonomic revisions (see Dai 1999). Despite this assumption, a great deal of variation in the morphology of the FV and its orientation among species of *Sinopotamon* have been described and illustrated (Chu *et al.* 2017a,b, 2018; Dai 1999). Ji *et al.* (2016) reconstructed the history of evolutionary diversification of *Sinopotamon* based on time-calibrated molecular phylogenetic analyses. Those authors found that a large number of species of *Sinopotamon* have diversified rapidly and recently. This phylogenetic dataset offers an ideal means to test morphological divergence of the genitalia, and to explore the evolutionary drivers that are responsible for genital evolution and rapid species diversification within the lineages of *Sinopotamon*.

Here we investigated the major driving forces responsible for the rapid divergence and coevolution of the genitalia in *Sinopotamon*. First, we used a geometric morphometric approach to detect whether shape variation of the genitalia of both sexes (G1 and FV) was associated with directional selection by comparing the results with those from 2 non-reproductive (non-genital) characters of these crabs that are involved in feeding (the third maxilliped merus [TMM] and ischium [TMI]). Second, we compared variation in the male and female genitalia between groups of species on different clades within *Sinopotamon* (Ji *et al.* 2016). Finally, we tested whether the morphology of female and male genitalia have coevolved by reconstructing the ancestral groundplans of the genitalia and plotting phenotypic divergence through time based on the temporal and spatial frameworks that have been proposed for these lineages.

## MATERIALS AND METHODS

### Taxon sampling

Some 948 specimens representing 69 species of *Sinopotamon* (including an undescribed species, *Sinopotamon* sp. 1) were collected from 181 localities across the entire geographical range of the genus in China (Fig. 1, Table S1). Specimens were collected by hand between 2009 and 2016 and preserved in 95% ethanol. The taxonomic coverage within *Sinopotamon* represents 81.2% of the described species in this genus and includes 666 males (from all 69 species) and 282 females (from 64 species). The outgroup taxa comprised 17 male and 19 female specimens of *Parapotamon spinescens* De Man, 1907. We used the taxonomic system proposed by Dai (1999) because this is largely supported by the phylogenetic results of Ji *et al.* (2016), and we included the newly described species *S. baokangense* Chu *et al.* (2017a). The phylogeny of *Sinopotamon* by Ji *et al.* (2016) recovered 3 clades (Clades I, II and III). Clade I is a monophyletic rounded lobes group whose species are distributed in the Sichuan Basin and surrounding mountains (SBRL); Clade II exhibited evidence of an exceptionally rapid and recent diversification; and Clade III was localized in eastern China where it underwent species diversification. The entire species set was also investigated as a whole to clarify morphological variation within *Sinopotamon*, in addition to investigating Clades I-III separately.

### Photo capture

The 2D images of the genital traits (the FV, and the dorsal and ventral views of the left G1) and of the 2 non-reproductive traits (TMM and TMI) were obtained using a stereoscopic zoom microscope NIKON 1500 and a NIKON DS-Fi2X digital microscope CCD. A total of 2735 2D photos were collected. The 3D images of genitalia from Clade I (SBRL) were scanned with micro-CT (MicroXCT 400) and these structures were reconstructed using Amira 5.2.2 based on 3D image stacks obtained. The final images were created using Photoshop CS5 and Illustrator CS6 (Adobe).

### Acquisition of geometric morphometric data

The software tpsUtil Ver.1.26 (Rohlf 2010) was used to convert the 2D images of the morphological structures (the FV, the dorsal and ventral views of the G1, TMM and TMI) into TPS files. The acquisition of geometric morphometric data was based on landmark-based methods (Adams & Rohlf 2000; Torres *et al.* 2013), with the knowledge that this method has the important limitation that it might not capture a sufficient number of landmarks to entirely understand the shape of a trait (Adams *et al.* 2004). Semi-landmarks based on the outline shape of a character provided a rich description of the shape of that character (Bai *et al.* 2015; Li *et al.* 2017; Orbach *et al.* 2017). This approach incorporated complete shape information regarding genital and non-reproductive traits that is not involved when using landmarks, and it has been proven to be suitable for solving complicated problems in morphological variation (Gunz *et al.* 2005; Perez *et al.* 2006). We captured all shape information of both the genital and non-reproductive body traits with a single curve (Fig. 2), and digitized the curve using tpsDig Ver.2.05 (Rohlf 2006). All curves were based on homologous or corresponding criteria. The curve for the FV was resampled into 25 semi-landmarks, while the curves for the dorsal and ventral views of the G1, TMM and TMI were resampled into 50, 50, 25 and 35 semi-landmarks, respectively. All configuration semi-landmarks were saved as the landmark dataset via tpsUtil (Rohlf 2010).

### Data analysis

Landmark data were analyzed using MorphoJ Ver.1.06d (Klingenberg 2011). Single data files of the morphological structures were imported into MorphoJ, and orthogonal projections conducted complete Procrustes fits to correct size and orientation. The shape differences of the morphological structures were assessed using principal component analysis (PCA) in MorphoJ. A scatterplot of the first 2 principal components (PC) was generated to summarize the distribution of individuals and the shape variations. The total variance of traits

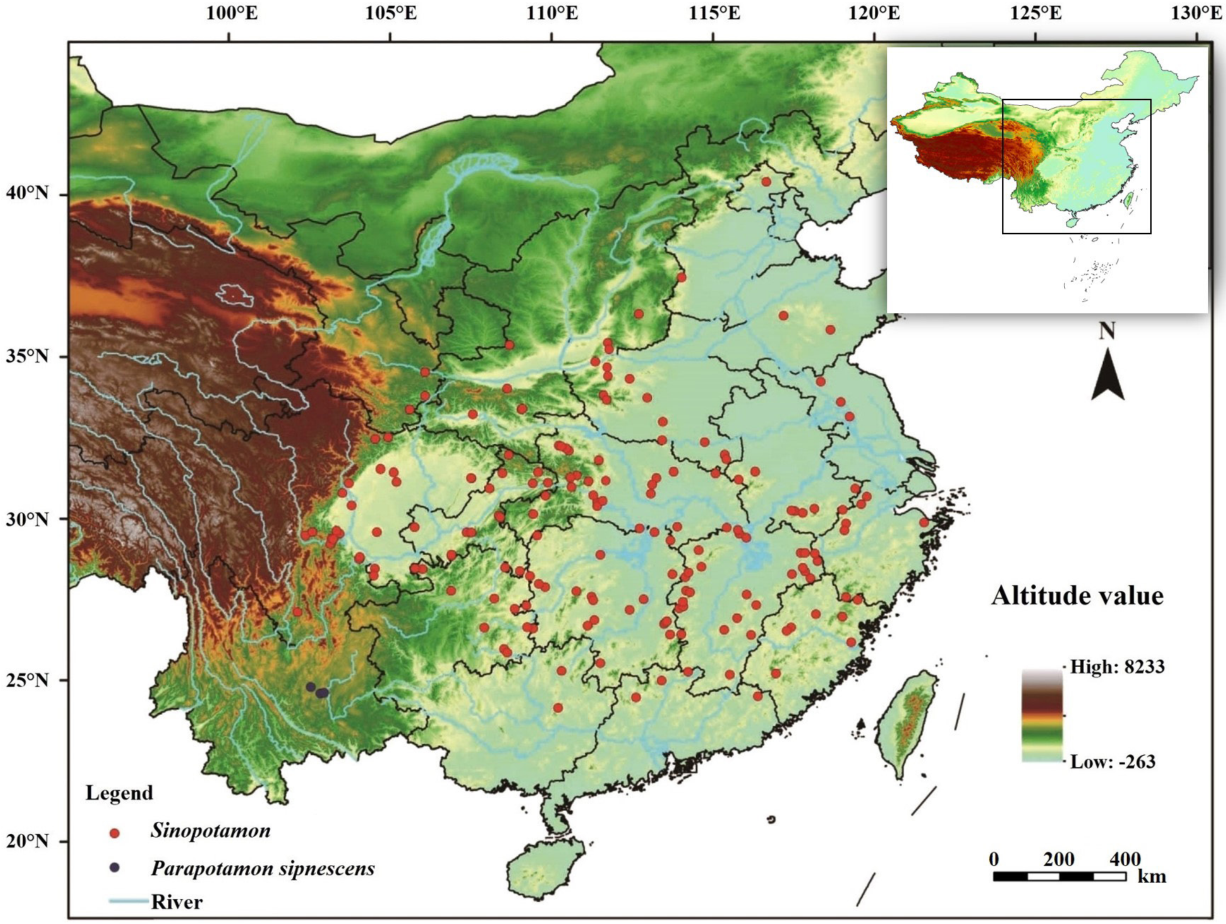

**Figure 1** Map showing the localities of the 69 species of *Sinopotamon* included in this study.

computed from the PCA was used for subsequent comparative analyses. After Procrustes superimposition, the covariance matrices were generated by MorphoJ. Subsequently, canonical variate analysis (CVA) was also conducted by MorphoJ for analyzing and testing differences among groups. Procrustes and Mahalanobis distances were calculated through the CVA analysis.

The aligned landmark data of 5 traits collected from the 60 ingroup species sampled in the phylogenetic tree reconstructed by Ji *et al*. (2016) were entered into MESQUITE Ver. 2.72 as a continuous matrix, and landmark data were associated with the molecular tree (including branch lengths and divergent times). The trace-all-characters and landmark-drawing modules of the RHETENOR package in MESQUITE were used to reconstruct the ancestral state of all nodes of the reproductive and non-reproductive traits. The shape of each ancestral node was shown using thin-plate splines. The ancestral state of each node was estimated and displayed. The data from each were then combined with the divergence time; the total variance of 3 Ma, 2 Ma, 1 Ma, 0.7 Ma and present, were calculated; and the trend through time was plotted. The information calculated for the nodes was arranged with the original landmark data for the traits from the 60 species of *Sinopotamon*, and the outgroups in EXCEL. PCA was used to infer the total variance of the traits among extant and extinct *Sinopotamon* in MorphoJ. Subsequently, Procrustes and Mahalanobis distances were calculated through the CVA analysis for examining and testing differences in the genital and non-reproductive traits.

## RESULTS

### Principal component analysis and canonical variate analysis results

The genital and non-reproductive morphological shapes were quantified using the Procrustes fit data and PCA based on 69 species of *Sinopotamon* and an outgroup species (*P. spinescens*). Of these, 948 were specimens of *Sinopotamon* (666 ♂ and 282 ♀), and 36 specimens were from the outgroup taxa (17 ♂ and 19 ♀). The shape variation of genital traits (the FV, and the dorsal and ventral views of the G1) and non-reproductive traits (TMM and TMI) were described by the first 2 PC, which were used for subsequent analyses (Fig. 3). The given probability level of 90% (nearly 90% of the data points; Fig. 3) presented the shape differences of traits via equal-frequency ellipse.

The first 2 PC of the FV, the dorsal and ventral views of the G1, TMM and TMI from *Sinopotamon* and the outgroup taxa accounted for 87.92%, 68.69%, 72.50%, 50.74% and 62.31% of the variation, respectively. The shape variation of both genital and non-reproductive traits was clearly separated for ingroup and outgroup species (Fig. 3a–e). The 2 non-reproductive traits overlapped in both ingroup and outgroup species (Fig. 3d and e). CVA revealed differences in genital and non-reproductive traits between *Sinopotamon* and *P. spinescens*. Mahalanobis and Procrustes distances showed significant differences among the ingroup and outgroup species analyzed (Tables S2 and S3, respectively). The

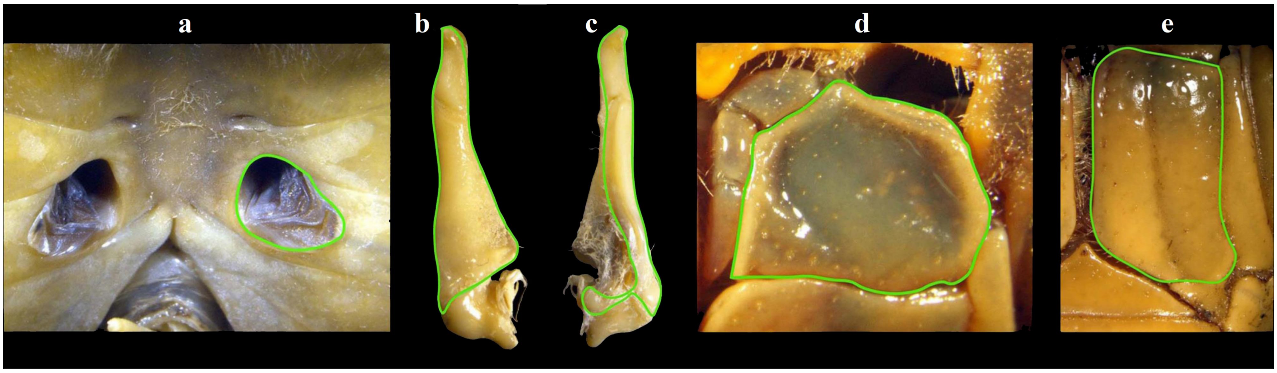

**Figure 2** Morphological traits used in this study. The green lines show the morphological traits that were captured based on geometric morphometric approaches. (a) female vulvae (FV); (b) male first gonopod (G1) dorsal view; (c) male first gonopod (G1) ventral view; (d) left third maxilliped merus (TMM); (e) left third maxilliped ischium (TMI).

*P*-values of Mahalanobis and Procrustes distances that were obtained with permutation tests (10 000 permutation rounds) were all smaller than 0.0001 (except for the TMM and TMI of *P. spinescens*), which confirmed significant differences in these results.

### Morphological variability between the genital and non-reproductive traits within species of *Sinopotamon*

Comparisons of the genital and non-reproductive traits for the entire group of species found rapid divergence of the genitalia in *Sinopotamon*. The largest shape variation was found in the FV, not only for the entire group (total variance, hereinafter referred to as Tv = 0.01409181) but also for Clades I, II and III (Tv = 0.01460892, 0.01159978 and 0.01289458, respectively). The morphological variance of the traits of the G1 and FV of the entire group (and of each of the clades) was significantly greater than those for the non-reproductive traits calculated by Platts distance and Mahalanobis distance (TMM and TMI). Furthermore, divergence in the FV traits was almost twice as high as in the G1 traits for the entire group, and divergence in the FV traits was almost 4 times as high as in the G1 traits for Clade I (SBRL) (Fig. 4).

### Shape variation and ancestral reconstruction of the genital and non-reproductive traits of *Sinopotamon* crabs

The variation of the genital and non-reproductive structures in the first 2 PC of all species of *Sinopotamon* is shown in Figure S1. Analyses of morphological variations of the entire group (and of each of the clades) indicated that PC1 and PC2 clearly described the changes of the FV from round and high (Clade I) to wide and flat (Clade II and Clade III) (Fig. 5). In Clade I, the G1 is thin and bends outward, whereas in Clades II and III the G1 is thick and bends inward (Fig. S1b and c and Fig. 5); non-reproductive traits showed no significant differences among Clades I–III (Fig. S1d and e). The

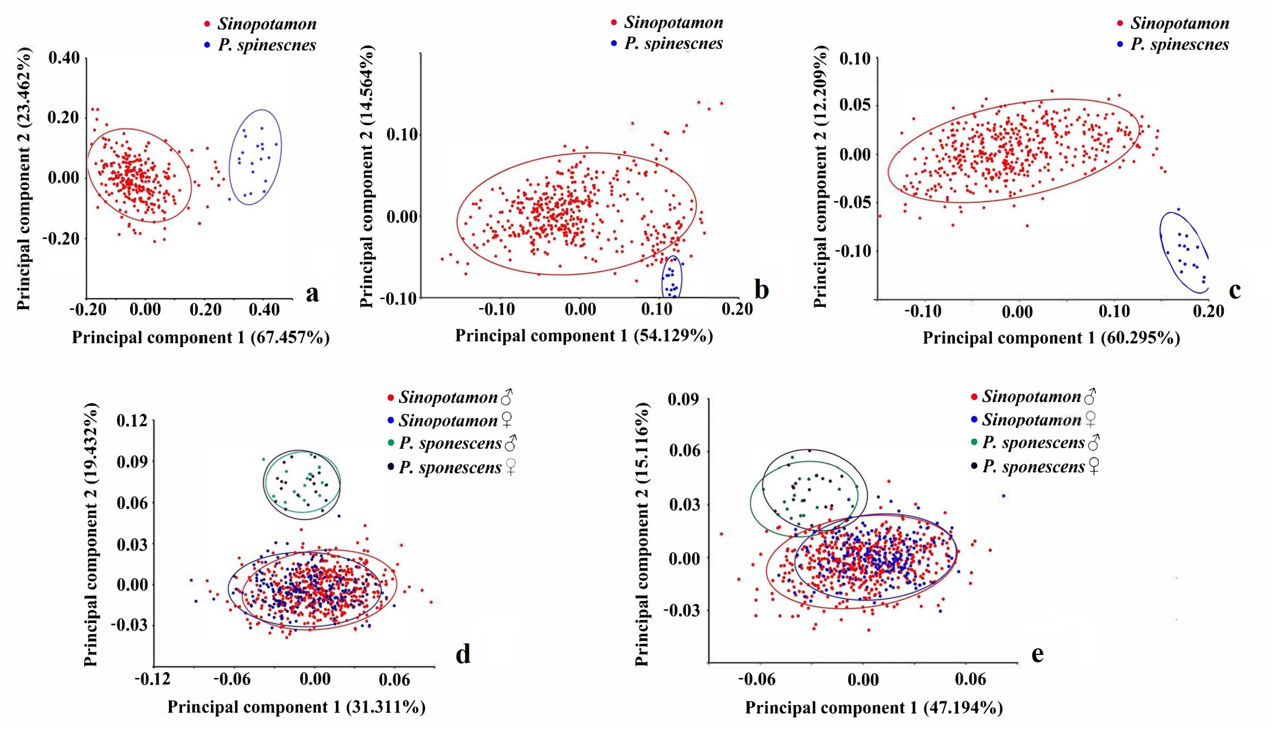

**Figure 3** Shape differences of the traits between *Sinopotamon* and *P. spinescens* based on principal component analysis, approximately 90% of the data points shown contain 90% equal frequency ellipses. (a) female vulvae (FV); (b) male first gonopod (G1) dorsal view; (c) male first gonopod (G1) ventral view; (d) third maxilliped merus (TMM); (e) third maxilliped ischium (TMI).

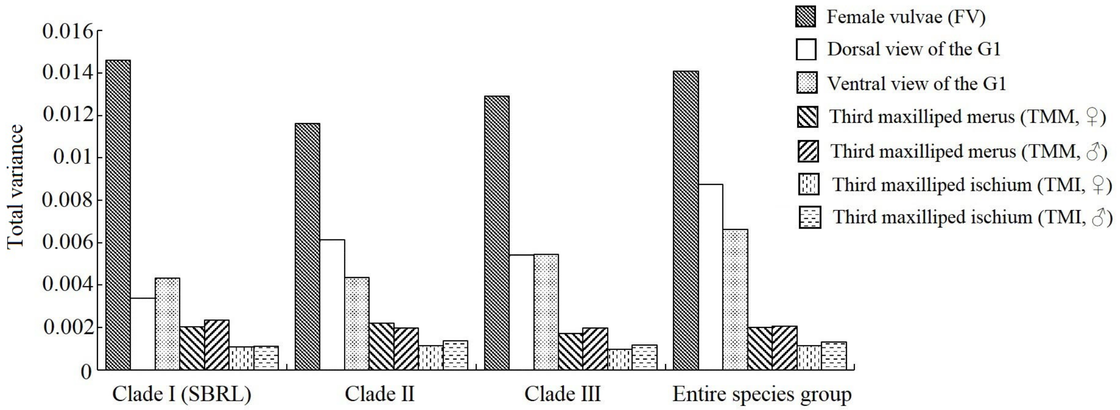

**Figure 4** Comparison of the total variance between the reproductive traits (FV, column 1; G1, columns 2–3) and non-reproductive traits (third maxillipeds, TMM and TMI, columns 4–7). The 7 columns on the left show the results for species in Clade I (G1 has a rounded terminal article) that are endemic to the Sichuan Basin and surrounding mountains (SBRL); the next 7 columns show the results for species in Clade II; the next 7 columns show the results for species in Clade III; and the 7 columns on the right show the results for the entire species group (all 69 species of *Sinopotamon*).

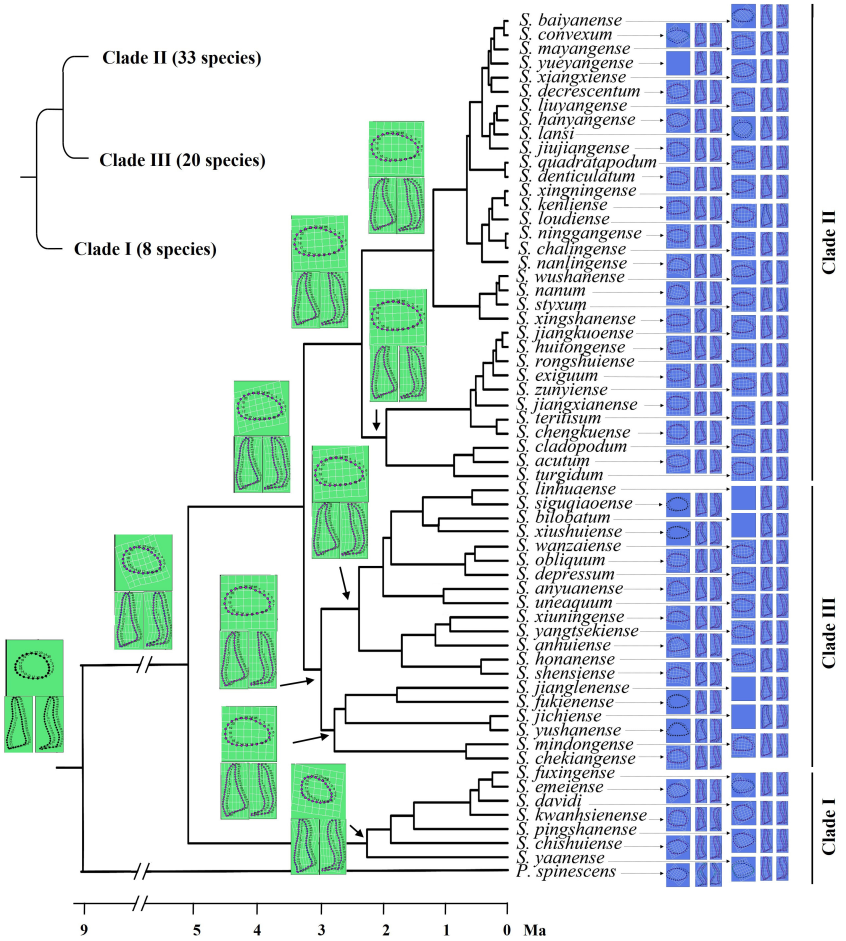

**Figure 5** The ancestral forms reconstruction of the genital traits within *Sinopotamon* and outgroup taxa. The splines show variation of the traits relative to the reference configuration. The shapes in green frame represent the ancestral forms on the phylogenetic tree following Ji *et al*. 2016, and the shapes in blue frame represent the measured genital traits (missing data left empty). The upper left corner shows a schematic representation of 3 clades and the number of species.

ancestral groundplans of the genital and non-reproductive traits of all nodes were reconstructed by linking the landmark data with the phylogenetic tree. The ancestral form of the genitalia in Clade I was quite different from that of the other 2 clades. In Clade I, the FV has revolved clockwise (round and high), while the terminal article of G1 has correspondingly become bent outward and thinner, while in Clades II and III, the FV has revolved counterclockwise (wide and flat) and the terminal article of G1 has correspondingly become curved inward and thicker (Fig. 5). The genital traits of species of *Sinopotamon* have, therefore, coevolved, and the variance of the current morphological traits is consistent with the ancestral state, but the degree is more significant (Fig. 5). The ancestral states of the non-reproductive body traits TMM and TMI in Clades I–III (node 4 in Fig. S2a and b) were found to be not significantly different from the common ancestor of *Sinopotamon* and from the outgroup taxa (Fig. S2a and b).

The temporal and spatial framework of the internal nodes within the molecular phylogenetic tree of *Sinopotamon* by Ji *et al.* (2016) was used to plot the total variance of the traits through time for the lineages in the phylogeny that existed at that particular time period (Fig. 6). This indicated that the genital traits (the FV, and the dorsal and ventral traits of the G1) followed a consistent trend in phenotypic divergence during evolutionary time, with a rapid shift during the Pleistocene (approximately 0.7 Ma). This recent shift in phenotypic divergence shown by the FV traits was significantly more rapid than the changes in G1 traits.

### DISCUSSION

This study is one of the first to quantify morphological variation in the male and female genitalia (the G1 and FV) among the vast majority of species of *Sinopotamon* using geometric morphometric statistical data, and comparisons of morphological variability between genital and non-reproductive structures. Our comparisons of morphological divergence of female and male genitalia in 4 subgroups of species within *Sinopotamon* provide the first evidence that variability of the shape of the G1 and FV of *Sinopotamon* is significantly greater than in the non-reproductive structures such as TMM and TMI. We also found that the FV traits are significantly more variable than male G1 traits, and that the FV and G1 covaried (as demonstrated by our 2D shape variation plotted through time combined with the ancestral state reconstructions of traits). This comparative evidence has revealed rapid evolutionary divergence and coevolution of the genitalia in male and female *Sinopotamon*, which may have contributed to rapid speciation in this genus.

This conforms with the expectation that genital struc-

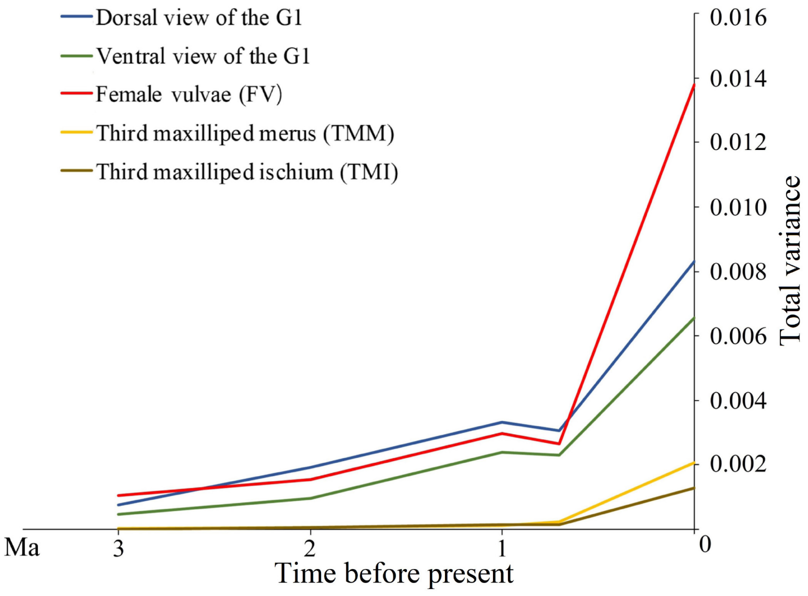

**Figure 6** Comparison of the total variance of the reproductive traits (FV, red line; G1 dorsal, blue line; G1 ventral, green line) and non-reproductive traits (third maxillipeds, TMM, yellow line; and TMI, brown line) through time for *Sinopotamon* in China.

tures are more likely to be divergent than are non-reproductive structures among closely-related species (Arnqvist & Thornhill 1998; Eberhard 1985, 2010; Kuntner *et al.* 2009). The rapid phenotypic divergence of the G1 and FV in *Sinopotamon* demonstrated by our quantitative geometric morphometric data suggests that it may be genetic isolation following widespread dispersal within a varied habitat that is driving the high speciation rate observed over a relatively short period of time in *Sinopotamon*. Furthermore, we demonstrated that the FV have undergone a cryptic rapid shape divergence that is faster than the rate of divergence in the G1. This phenomenon has been overlooked in previous studies of *Sinopotamon* that considered the FV to be simple invariant structures that were not useful in taxonomic studies (see Dai 1999). Rapid divergence in genitalia driven by a common persistent directional selective force has led to diversification within lineages (Puniamoorthy *et al.* 2010; Kwan *et al.* 2013). In addition, coevolution of the genitalia has promoted reproductive isolation, resulting in the rapid evolution of complementary genitalia within populations/species (Evans *et al.* 2011; Kraaijeveld *et al.* 2011). Nevertheless, a complex coevolved arrangement has yet to be demonstrated in freshwater crab biology. Guinot *et al.* (2013) suggested that changes in the morphology of male genitalia coevolved with the female genitalia, at least in marine crabs. The correlated divergence in the G1 and FV during the evolutionary history of the genitalia of species of *Sinopotamon* recovered by our quantitative analyses, and by our ancestral state reconstructions of genital traits with plotting total variance through time, indicates that genitalia in male and female species have coevolved. Variation in the morphology of the genitalia is the product of coevolution, which prevents hybridization and enhances reproductive isolation (Brennan & Prum 2015; Eberhard 1985). This, in turn, promotes genetic isolation, divergence and speciation, and has undoubtedly contributed to the diversification of this fauna.

Ji *et al.* (2016) proposed that past climate change during the Pleistocene (approximately 0.7 Ma) may have accelerated the rate of diversification in *Sinopotamon*. In the present study we provided new evidence from comparative geometric morphometrics that further demonstrated the rapid divergent evolution of genital traits that occurred during the period of recent rapid radiation in the Pleistocene (approximately 0.7 Ma). The morphological changes in genitalia in *Sinopotamon* are the result of the effects of coevolution between the male and female reproductive systems to bring about successful copulation and spermatophore transfer. The results are consistent with the supportive evidence obtained in previous works on millipedes (*Antichiropus*) (Wojcieszek & Simmons 2011) and water striders (Rowe & Arnqvist 2012) that have driven genital divergence.

The rapid genital diversification of *Sinopotamon* and the coevolution of male and female genitalia demonstrated here is also mediated by the exposure of these crabs to novel ecological conditions. However, while cases of genital diversification are widely documented in macroevolution in higher taxa (e.g. Takami & Sota 2007; Martin 2012; Orbach *et al.* 2017) this phenomenon has rarely been studied between species of the same genus. Sexual selection in freshwater crabs may not involve the external genitalia (because these are normally covered over by pleon in both sexes, and, therefore, the genitalia are not directly exposed to the selective forces operating in the environment). Instead, it may be the enlarged major cheliped of adult male freshwater crabs that signals the fitness of the male to females, and that influences mate choice in this group. In contrast, the smaller minor cheliped of the adult male is not remarkably enlarged and is similar in size to the chelipeds of adult female crabs, and, as such, may simply play a role in feeding (Guinot *et al.* 2013).

In summary, our results highlight the temporal and spatial evolution of genitalia in both sexes among a large number of species of the most diverse genus of freshwater crabs found in China. We provide robust evidence for the rapid divergence of female genitalia (which is an original finding that is sure to prompt further investigations), and for the coevolution of the male and female genitalia of freshwater crabs, which can help to establish an ideal model system for the study of the evolution of genitalia among other groups of brachyurans.

### ACKNOWLEDGMENTS

We thank Professor Kaiya Zhou (College of Life Sciences, Nanjing Normal University) and Professor Naifa Liu (School of Life Sciences, Lanzhou University) for invaluable comments on this study. We thank colleagues, including Professor Hongxin Niu, for assistance in sampling collections, (College of Life Sciences, Henan Normal University), Professor Zhonghu Liu (School of Life Sciences, Henan Agriculture University), Professor Zuhao Huang (School of Life Sciences, Jinggangshang University), Professor Xiaoping Yu (School of Life Sciences, Shanxi Normal University) and Dr Xiaoli Wang and Wangwang Yang (School of

Life Sciences, Lanzhou University). We also thank Professor Changfa Zhou, Dr Jianhua Dai, Dr Yufang Sun, Dr Fang Fang, Dr Yongkun Ji, Dr Kelin Chu, Juanjuan Xu, Lin Zhu, Cha He, Jiajia Cheng, Dehua Zhang, Guanghui Qian, Guangyue Wang and Danhong Song (College of Life Sciences, Nanjing Normal University) for assistance with collecting samples, Dr Yongkun Ji for providing molecular phylogenetic data, Xiaorui Liu (College of Life Sciences, Nanjing Normal University) for providing morphological photos, and Shengjin Xie (Henan Polytechnic University) for helping with the morphological data analysis. This project received funding (to Hongying Sun) from the the National Natural Science Foundation of China (No. 31471972 and 31772427). We are grateful to two anonymous reviewers and editors for their constructive comments and suggestions that substantially improved the quality of this manuscript.

### SUPPLEMENTARY MATERIALS

**Cite this article as:**

## Cite this article as:

Yao F, Shi B, Wang X *et al*. (2020). Rapid divergent coevolution of freshwater crab genitalia facilitates a burst of species diversification. *Integrative Zoology* **15**, 174–86.

## Supporting information

Additional supporting information may be found in the online version of this article at the publisher's website.
**Table S1** Localities and number of specimens of the 69 species of *Sinopotamon* used in this study.
**Table S2** Shapes difference in the genital traits between *Sinopotamon* and *P. spinescens* crabs (*P*‐values are on the right; distances are on the left).
**Table S3** Shape difference in the general traits between *Sinopotamon* and *P. spinescens* crabs.
**Figure S1** Shape variation of the reproductive traits (FV, female vulva; G1, first gonopod) and non‐reproductive traits (TMI, left third maxilliped ischium; TMM, third maxilliped merus and ischium) described by the first 2 principal components.
**Figure S2** Reconstruction of ancestral forms of the non‐reproductive traits of the third maxillipeds in Sinopotamon and the outgroup taxa.

Supporting Information
