# Supplementary material for: Rapid divergent coevolution of Sinopotamon freshwater crab genitalia facilitates a burst of species diversification
Source: Integr Zool. 2020 Apr 22;15(3):174–86. doi: 10.1111/1749-4877.12424 (PMC7216907; doi:10.1111/1749-4877.12424)
Supplement: Supplementary file 1 — Supporting Information [file INZ2-15-174-s001.pdf]

doi: 10.1111/1749-4877.12424

SUPPLEMENTARY MATERIALS

Table S1 Localities and number of specimens of the 69 species of *Sinopotamon* used in this study

Species	Localities	Number (♂/♀)
Ingroup		
S. acutum	Suining, Sichuan	3/1
	Kai County, Chongqing	9/1
	Luoyang, Henan	7/0
	Shangluo, Shaanxi	6/0
	Mianyang, Sichuan	4/1
	Fuling County, Chongqing	1/2
	Wangsheng District, Chongqing	1/2
	Tianshui, Gansu	3/1
	Xi'an, Shaanxi	1/1
	Yichang, Hubei	2/4
	Dazhou, Sichuan	2/2
	Dazu County, Chongqing	1/0
	Shizhu County, Chongqing	1/0
S. anhuiense	Chizhou, Anhui	5/1
S. anyuanense	Ganzhou, Jiangxi	15/0
	Lechang, Guangdong	3/1
	Nanxiong, Guangdong	3/2
	Mei County, Guangdong	5/0
	Qingyuan, Guangdong	5/4
	Meizhou, Guangdong	4/0
S. baiyanense	Longshan County, Hunan	6/1
S. baokangense	Baokang County, Hubei	3/2
S. bilobatum	Wanzai County, Jiangxi	5/0
S. chalingense	Yanling County, Hunan	5/0
	Chaling County, Hunan	2/2
S. changanense	Xianyang, Shaanxi	4/1

Species	Localities	Number (♂/♀)
S. chekiangense	Ningbo, Zhejiang	1/0
	Chun'an County, Zhejiang	7/3
	Anji County, Zhejiang	1/1
S. chengkuense	Chengkou County, Chongqing	10/2
S. chishuiense	Chishui, Guizhou	4/1
S. cladopodum	Chongzhou, Sichuan	3/1
	Xinjin County, Sichuan	4/3
	Dujiangyan, Sichuan	3/0
	Mianyang, Sichuan	4/0
S. convexum	Xiushan County, Chongqing	2/1
	Youyang County, Chongqing	2/0
S. davidi	Yidu, Hubei	4/7
	Yichang, Hubei	1/0
	Yichang, Hubei	1/0
	Dazhou, Sichuan	3/2
	Guangyuan, Sichuan	6/1
	Neijiang, Sichuan	5/1
	Yibin, Sichuan	5/7
	Mianyang, Sichuan	0/1
	Wansheng District, Chongqing	3/3
	Shizhu County, Chongqing	4/1
	Fuling County, Chongqing	4/2
S. decrescentum	Songzi, Hubei	9/2
	Yidu, Hubei	10/1
S. denticulatum	Jianshi County, Hubei	1/7
	Jingshan County, Hubei	5/4
S. depressum depressum	Dexing, Jiangxi	1/0
	Wuyuan County, Jiangxi	5/0
	Shitai County, Anhui	2/0
	Tian Tang Zhai Scenic Spot, Anhui	5/1
S. depressum	Xinyang, Henan	4/4

Species	Localities	Number (♂/♀)
shangchengense		
	Enshi, Hubei	2/2
	Macheng, Hubei	1/1
S. ebianense	Ebian County, Sichuan	1/2
S. emeiense	Emeishan, Sichuan	15/8
S. exiguum	Shiqian County, Guizhou	9/10
S. fukienense	Fuzhou, Fujian	4/1
	Pingnan County, Fujian	4/1
	Longyan, Fujian	2/2
	Yiyang County, Jiangxi	3/0
	Shangrao, Jiangxi	2/0
	Xingguo County, Jiangxi	1/0
	Jian'ou, Fujian	4/1
	Jiangle County, Fujian	1/0
	Yihuang County, Jiangxi	2/1
	Chongren County, Jiangxi	1/0
	Ningdu County, Jiangxi	1/1
	Yongfeng County, Jiangxi	2/0
	Qingyuan County, Zhejiang	3/1
S. fuxingense	Chishui, Guizhou	4/1
	Pingshan County, Sichuan	2/1
	Yibin, Sichuan	1/3
S. hanyangense	Shishou, Hubei	1/2
	Chibi, Hubei	6/4
	Changde, Hunan	2/1
	Tongcheng County, Hubei	1/0
	Xiangtan County, Hunan	1/0
S. honanense	Changzhi, Shanxi	3/0
	Pingdingshan, Henan	9/1
	Zhumadian, Henan	2/0

Species	Localities	Number (♂/♀)
	Xiaogan, Hebei	3/1
	Ruyang County, Henan	1/2
S. huitongense	Huaihua, Hunan	0/2
	Yuping County, Guizhou	2/1
S. jiangkuoense	Huaihua, Hunan	4/4
	Jinping County, Guizhou	4/1
	Shaoyang, Hunan	0/2
S. jianglenense	Jiangle County, Fujian	3/0
S. jiangxianense	Yuncheng, Shanxi	6/2
	Jingxing County, Hebei	5/2
S. jixiense	Linan, Zhejiang	1/0
S. jiujiangense	Jiujiang, Jiangxi	3/2
	Jiujiang, Jiangxi	2/0
S. kenliense	Kaili, Guizhou	1/9
S. kwanhsiense	Zigui County, Hubei	2/1
S. lansi	Shangrao, Jiangxi	2/3
	Tianmen, Hubei	5/1
	Guilin, Guangxi	1/1
	Yushan County, Jiangxi	3/0
	Zhuzhou, Hunan	2/0
	Yichun, Jiangxi	4/0
S. linhuaense	Lianhua County, Jiangxi	7/0
S. liuyangense	Liuyang, Hunan	10/2
	Yichun, Jiangxi	1/2
S. loudiense	Hengyang, Hunan	2/2
	Loudi, Hunan	2/3
	Shaoyang, Hunan	3/0
S. mayangense	Mayang County, Hunan	25/4
S. mindongense	Qingyuan County, Zhejiang	2/0
	Shouning County, Fujian	6/2

Species	Localities	Number (♂/♀)
S. nanlingense	Yongzhou, Hunan	7/2
	Fenghuang County, Hunan	8/2
S. nanum	Kai County, Chongqing	11/2
	Wuxi County, Chongqing	1/1
	Fengjie County, Chongqing	1/0
S. ninggangense	Jinggangshan, Jiangxi	4/1
S. obliquum	Xiushui County, Jiangxi	1/1
	Lushan Mountain, Jiangxi	1/0
S. pingshanense	Pingshan County, Sichuan	9/3
	Pingshan County, Sichuan	6/1
S. planum	Yangzhou, Jiangsu	1/0
	Beijing	2/2
	Yishui County, Shandong	2/1
	Tai'an, Shandong	1/0
S.quadratapodum	Enshi, Hubei	1/1
S. rongshuiense	Rongjiang County, Guizhou	2/3
	Congjiang County, Guizhou	3/1
	Jinxiu Yao Autonomous County, Guangxi	8/1
S. shensiense	Longnan, Gansu	0/1
	Hanzhong, Shanxi	5/0
	Nanyang, Henan	1/0
	Shiyan, Hubei	1/0
	Zhushan County, Hubei	1/0
	Longnan, Gansu	3/1
	Yuanqu County, Shanxi	4/0
	Shangluo, Shaanxi	2/0
S. siguqiaoense	Shangrao, Jiangxi	3/2
S. styxum	Badong County, Hubei	4/6
S. teritisum	Fang County, Hubei	2/0
	Zhushan County, Hubei	4/6

Species	Localities	Number (♂/♀)
S. turgidum	Ruichang, Jiangxi	2/1
	Zhijiang, Hubei	7/4
S. unaequum	Luxi County, Jiangxi	2/6
S. wanzaiense	Wanzai County, Jiangxi	1/1
	Luxi County, Jiangxi	1/0
S. wushanense	Wushan County, Chongqing	5/1
S. xiangxiense	Jishou, Hunan	2/3
	Xupu County, Hunan	3/4
S. xingningense	Xinning County, Hubei	1/2
S. xingshanense	Zigui County, Hubei	3/4
	Yichang, Hubei	1/0
S. xiuningense	Chun'an County, Zhejiang	13/2
S. xiushuiense	Yifeng County, Jiangxi	3/5
S. yaanense	Emeishan, Sichuan	2/4
	Emeishan, Sichuan	4/4
	Emeishan, Sichuan	3/0
	Ya'an, Sichuan	1/5
S. yangtsekiense shanxianense	Shan County, Henan	9/10
	Sanmenxia, Henan	3/0
S. yangtsekiense tongbaiense	Xinyang, Henan	6/0
	Tongbai County, Henan	5/2
	Jingshan County, Hubei	2/0
	Shangcheng County, Henan	3/0
	Xi County, Henan	4/0
S. yangtsekiense yangtsekiense	Lianyungang, Jiangsu	2/0
	Guangde County, Anhui	1/0
	Xuzhou, Jiangsu	2/0
	Lu'an, Anhui	3/0
	Anji County, Zhejiang	4/1

Species	Localities	Number (♂/♀)
	Luoning County, Henan	5/2
S. yixianense	Huangshan, Anhui	1/1
S. yueyangense	Chaling County, Hunan	6/0
S. yushanense	Yushan County, Jiangxi	2/1
S. zunyiense	Zuiyi, Guizhou	2/3
S. sp. 1	Jingzhou, Hubei	4/1
Total		666/282
Outgroup		
P. spinescens	Yuxi, Yunnan	9/15
	Fuxian Lake, Yunnan	2/0
	Chengjiang County, Yunnan	6/4
Total		683/301

S. sp. 1 is an undescribed species included in this study.

Table S2 Shapes difference in the genital traits between *Sinopotamon* and *P. spinescens* crabs (*P*-values are on the right; distances are on the left).

Shape divergence		Mahalanobis distance		Procrustes distance	
		Sinopotamon spp.	*P. spinescens*	*Sinopotamon* spp.	*P. spinescens*
Female vulvae (FV)	*Sinopotamon* spp.		<0.0001		<0.0001
	P. spinescens	10.2802		0.3981	
Dorsal view of the G1	*Sinopotamon* spp.		<0.0001		<0.0001
	P. spinescens	19.4929		0.1880	
Ventral view of the G1	*Sinopotamon* spp.		<0.0001		<0.0001
	P. spinescens	16.0252		0.2124	

Table S3 Shape difference in the general traits between *Sinopotamon* and *P. spinescens* crabs.

Shape divergence		Mahalanobis distance				Procrustes distance			
		Sinopotamon spp. ♂	*Sinopotamon* spp. ♀	*P. spinescens* ♂	*P. spinescens* ♀	*Sinopotamon* spp. ♂	*Sinopotamon* spp. ♀	*P. spinescens* ♂	*P. spinescens* ♀
Third maxilliped merus (TMM)	*Sinopotamon* spp. ♂		<0.0001	<0.0001	<0.0001		<0.0001	<0.0001	<0.0001
	Sinopotamon spp. ♀	0.9005		<0.0001	<0.0001	0.0120		<0.0001	<0.0001
	P. spinescens ♂	9.5788	9.4480		0.7002	0.0812	0.0800		0.911
	P. spinescens ♀	9.5525	9.4776	2.3975		0.0795	0.0784	0.0082	
Third maxilliped ischium (TMI)	*Sinopotamon* spp. ♂		<0.0001	<0.0001	<0.0001		<0.0001	<0.0001	<0.0001
	Sinopotamon spp. ♀	1.3990		<0.0001	<0.0001	0.0094		<0.0001	<0.0001
	P. spinescens ♂	10.691	10.4958		0.0056	0.0534	0.0571		0.1813
	P. spinescens ♀	10.1008	9.9905	5.6741		0.0525	0.0551	0.0115	

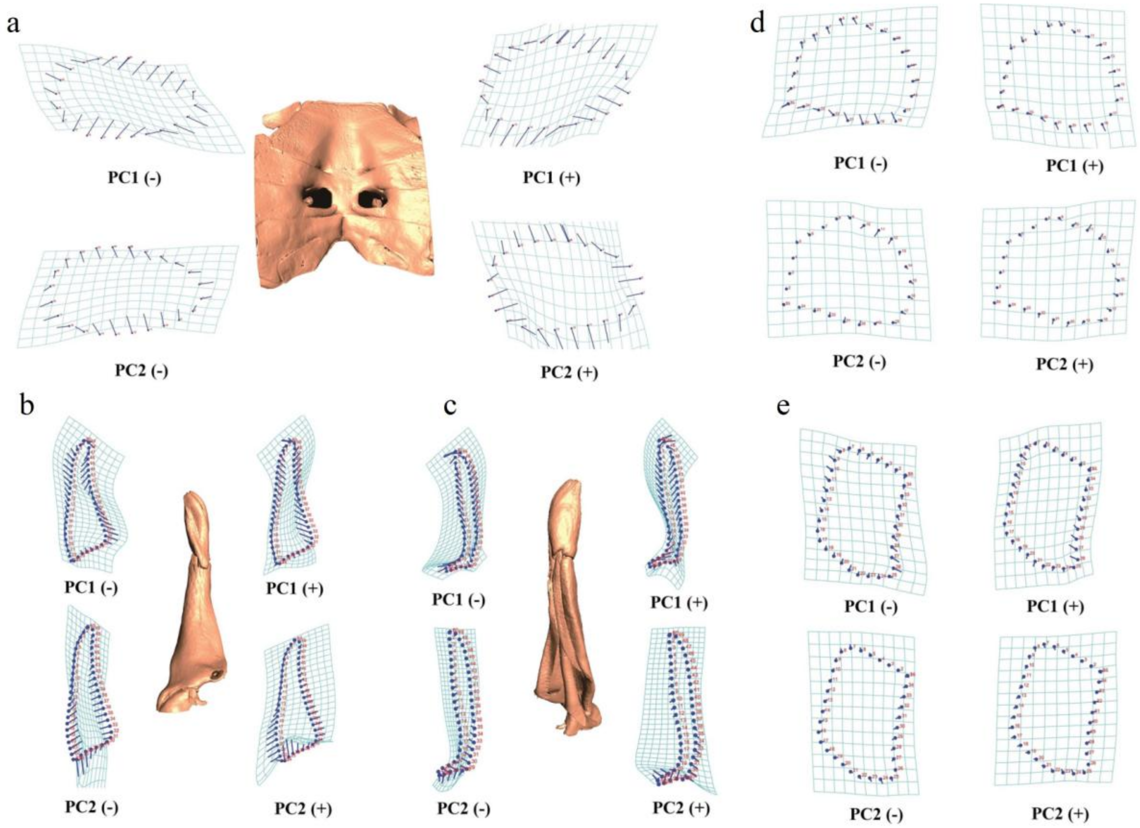

Figure S1 Shape variation of the reproductive traits (female vulva, FV and first gonopod, G1) and non-reproductive traits (third maxilliped merus and ischium, TMM and TMI) described by the first two principal components. The 3D image of FV and the dorsal and ventral views of left G1 showing in the figure. a, FV deformation grids. b, dorsal G1 deformation grids. c, ventral G1 deformation grids. c, TMM deformation grids. e, TMI deformation grids.

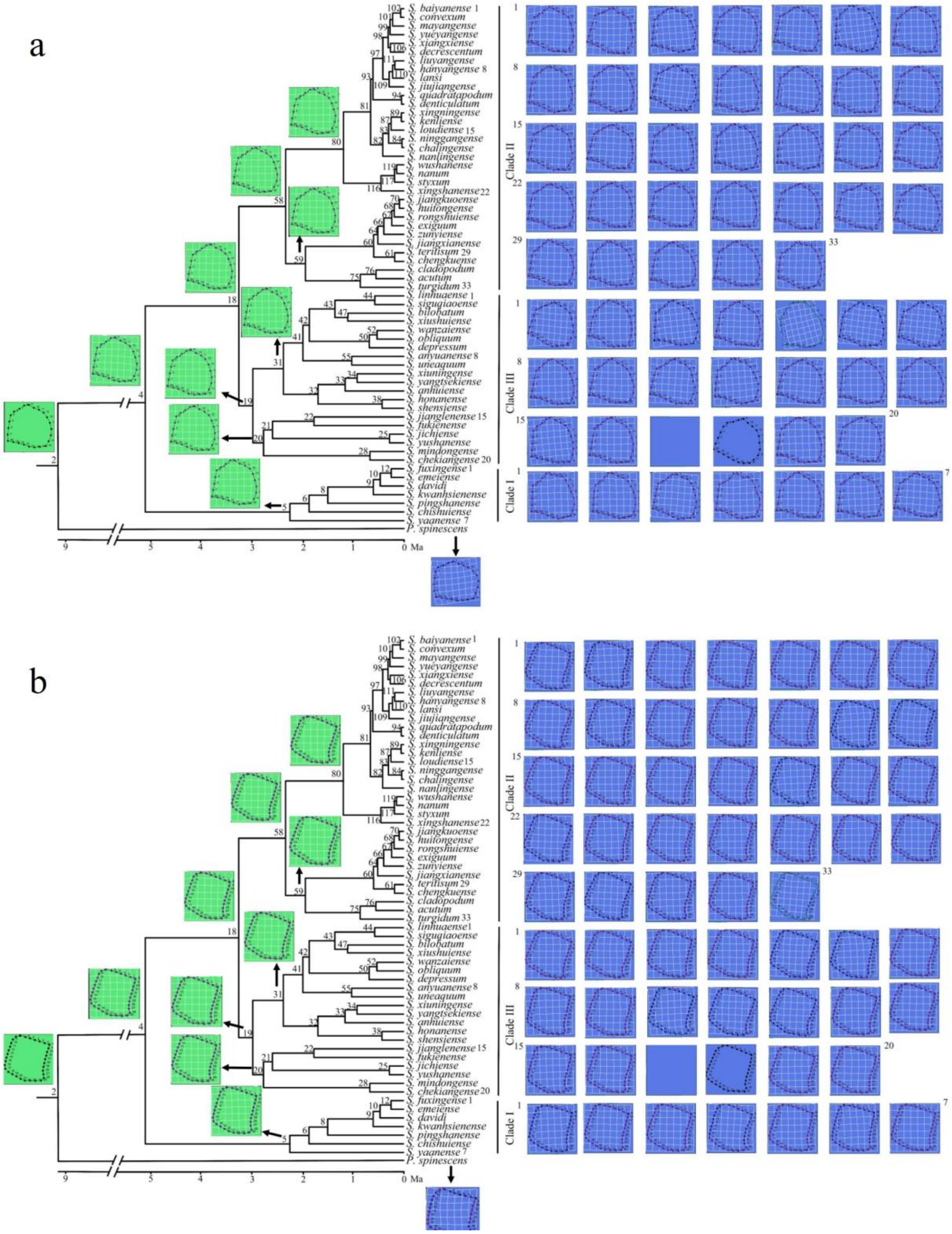

Figure S2 Reconstruction of ancestral forms of the non-reproductive traits of the third maxillipeds in *Sinopotamon* and the outgroup taxa. The splines indicate deformation of the shapes relative to the reference configuration. The green splines represent the ancestral forms and the blue splines represent the 60 species of *Sinopotamon*. The blue splines correspond to species on the phylogenetic tree from left to right, and the blank box represents a lack of data for the corresponding species. The phylogenetic tree was summarized and reconstructed from earlier molecular results (Ji *et al*. 2016). a, third maxilliped merus (TMM). b, third maxilliped ischium (TMI).
